# Green synthesis of fluorescent N-doped carbon quantum dots from castor seeds and their applications in cell imaging and pH sensing

**DOI:** 10.1038/s41598-024-78745-0

**Published:** 2024-11-13

**Authors:** Salah Elkun, M. Ghali, T. Sharshar, M. M. Mosaad

**Affiliations:** 1 Physics Department, Faculty of Science, Kafrelsheikh University, 33516 Kafr El-Sheikh, Egypt; 2 Institute of Basic and Applied Sciences, Egypt-Japan University of Science and Technology , 21934 New Borg Al-Arab, Egypt

**Keywords:** N-CQDs, Castor seeds, Cytotoxicity, Antimicrobial activity, pH sensor, Quantum dots, Sensors

## Abstract

Water-soluble fluorescent N-doped carbon quantum dots (N-CQDs) were hydrothermally prepared through a green synthesis route using castor seeds as a single precursor and a hydrothermal method. Several experimental techniques have been used to characterize synthesized N-CQDs to confirm their structure and to verify their applicability in cell imaging and pH sensing. The synthesized N-CQDs were found to have are characterized by amorphous nature with a spherical shape with an average particle size of 6.57 nm as revealed from XRD and TEM measurements. The FTIR results reveal the presence of carboxylic and hydroxyl functional groups on the surface of the CQDs, which was also confirmed by XPS analysis. The fluorescence characterization of the synthesized N-CQDs showed blue emission and excitation dependence with good photostability. It was found that the optimal excitation and emission wavelengths were (λ_Ex_ = 360) and (λ_Em_ = 432) nm, respectively. The fluorescence quantum yield (QY) of about 9.6% at the optimum excitation wavelength 360 nm. Moreover, the fluorescence intensity of N-CQDs showed good linear dependence with the pH values in ranges of 3.5 − 7.5 and 8 − 12 as well as high sensitivity for slight changes of pH values. According to these results, two fluorescent pH sensors were created based on acidic and basic media. The obtained N-CQDs have zeta potential of -21.86 mV and thus have excellent stability in water. Moreover, N-CQDs derived from the castor seeds have antimicrobial activity and exhibits low cytotoxicity to WI-13 cells with IC_50_ = 394.4 ± 13.8 µg/mL. The results of this study demonstrated that the synthesized N-CQDs derived from castor seeds can be used as pH sensing and antimicrobial materials. On the other hand, they are also promising in applications in cell imaging, thermo-sensing and optoelectronics.

## Introduction

Fluorescent carbon quantum dots (CQDs) are a new class of carbon-based nanomaterials, with a spatial size generally less than 10 nm. They have attracted great attention in many research directions due to their various properties^1,2^. Carbon quantum dots had environmental friendliness, broad absorption spectrum, tunable optical properties, good charge transfer/separation ability, high fluorescence and good quantum yield as well as various biological properties^3,4^. Therefore, they appear as promising materials for a wide range of applications such as optoelectronic applications^5^, fluorescent probes, pH sensor^6^, cell imaging^7^, photocatalysis^8^, optical thermometry^9^. However, due to their limited fluorescence quantum yields and few active sites for modification, many efforts have been made to enhance their luminescent properties. Doping with non-metallic heteroatoms has been demonstrated to be a practical way to improve the fluorescence quantum yields of CQDs^10^. Thanks to nitrogen’s similar atomic size to that of carbon and its electron donor character, the nitrogen atom becomes the natural candidate for doping in CQDs, and nitrogen doping has been demonstrated to enhance fluorescence quantum yields with significant advantages^11,12^. Besides, natural materials usually contain multifarious heteroatoms, so CQDs synthesized from these materials are always packed with various surface groups and retain unique properties without further modification. Therefore, many efforts have been earmarked to synthesize CQDs from various natural biomass/biowaste^7^. Nowadays, several methods have been developed to prepare fluorescent carbon quantum dots, such as solvothermal/hydrothermal treatment^13^, arc discharge^14^, laser ablation^2,15^, electrochemical^13^, ultrasonic^16^, and microwave^17^. Among these methods, hydrothermal processing is one of the best and low-cost green chemistry methods to prepare large-scale fluorescent CQDs using biomass resources as carbon sources^18^. In addition to, this method is easy to set up and produces uniformly shaped particles with high yield^19^. Therefore, efforts have been inspired to synthesize fluorescent CQDs using different natural resources such as castor seeds^9^. The castor seed plant (scientific name: *Ricinus communis*) was a popular ornamental plant in gardens due to its ability to produce large quantities of fertile seeds, which are dispersed effectively. Consequently, this plant has become an important environmental weed that is found in many diverse locations. The castor seeds contain the oil as well as the toxic protein ricin, therefore, these seeds are considered a rich source of organic compounds, that can used as carbon source^20^.

To our knowledge, the hydrothermal approach has not been previously used to synthesize N-CQDs from castor seeds. Moreover, castor seed-based CQDs have never been applied in pH sensing or cell imaging. The importance of pH is well known in a variety of fields, including biology, agriculture, industry, and the environment. In addition to everyday applications such as food and beverage analysis and disease detection, pH monitoring is essential for scientific research. To bypass the drawbacks of typical organic fluorescent dye, such as background autofluorescence and photobleaching, pH-sensitive nanoprobes based on organic dyes and nanocarriers have been created. However, since organic dyes and nanocarriers have large particle sizes and low biocompatibility, it is still difficult to provide long-term pH monitoring without biological damage^21^. Carbon quantum dots with small size, excellent photostability and good biocompatibility have the potential to provide solutions for pH sensing in many fields especially pharmaceutical and in vivo medical applications^6^.

Bioimaging refers to a non-invasive technique to visualize a biological system in real time. It includes the study of subcellular arrangement and whole cells as well as tissues and multicellular organisms. CQDs have been identified as attractive candidates for cell imaging due to their greater tunable photoluminescence (PL), lower toxicity, hydrophilic feature, and great photochemical stability under UV illumination. Most CQDs effectively generate blue emission with short excitation spectra, which severely limits bioimaging. Fortunately, several studies have shown that heteroatom doped such as N-CQDs have extended wavelengths and multicolor fluorescent, making them suitable for real-time and longer-time cell imaging^22^.

The main objective of the present work is to synthesize the N-CQDs using a hydrothermal method and castor seeds as the sole carbon source. One goal is to characterize CQDs prepared using different experimental techniques and systematically examine the fluorescence of the N-CQDs for their potential for applications in pH sensing and cell imaging. As one of the objectives, cytotoxicity and antimicrobial activity measurements of the synthesized N-CQDs will be performed to enhance the advantages of their applications.

## Experimental details

### Synthesis of N-CQDs

**Fig. 1:**
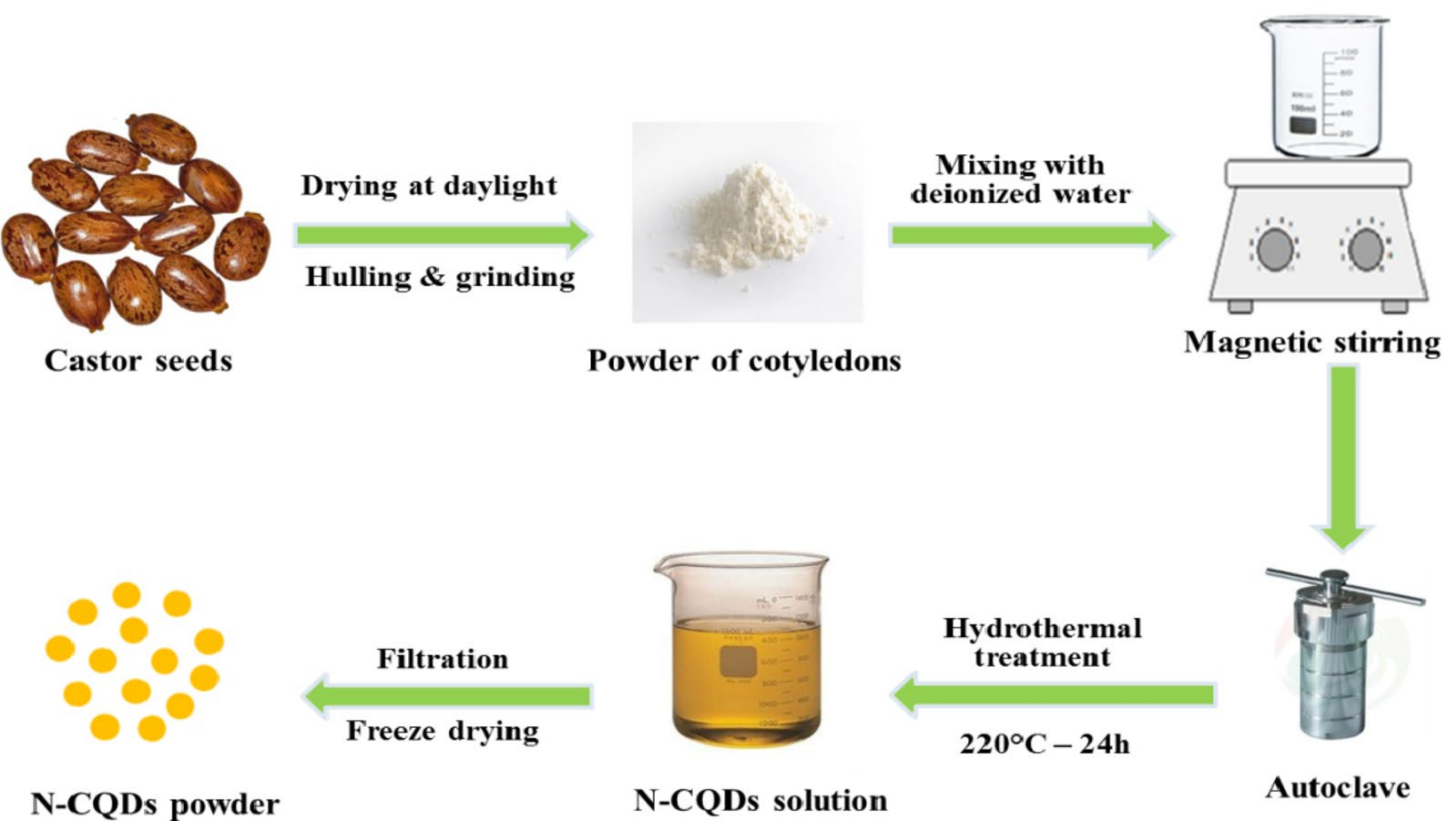
Scheme for synthesis of carbon quantum dots (N-CQDs) from castor seeds though a simple hydrothermal method.

N-doped fluorinated carbon quantum dots were synthesized from castor seeds though a simple hydrothermal treatment process, which is shown in Fig. 1. Initially, castor seeds were obtained from local market in Egypt and dried under room temperature, after which they were ground and homogenized. 0.5 g castor seed powder was mixed homogeneously with 50 mL of deionized water. The solution was stirred for 1 h at room temperature, then transferred to a Teflon-lined stainless-steel autoclave, and heated at 220^°^C for 24 h. After the reaction was completed, the yellowish-brown transparent product was cooled naturally at room temperature. Insoluble macroparticles were filtered using a 0.22 μm filter membrane and dialyzed using a dialysis bag (1 kDa) for 24 h under continuous magnetic stirring against 200 mL deionized water to remove reaction residues and byproducts. The resulting solution of deionized water with dispersed N-CQDs was stored at a temperature of < 4^º^C. Finally, a portion of the N-CQDs solution was vacuum freeze-dried to obtain the solid brown powder product. This powdered product has been used for further characterization and applications.

An X-ray diffractometer (XRD-6100, Shimadzu, Max power 3 kW, 2θ range from − 6˚ to 163˚, Japan) with Cu-K_α_ radiation (λ = 0.15418 nm) was used to measure the XRD pattern of the as-prepared N-CQDs. The synthesized N-CQDs were characterized using Fourier transform infrared spectroscopy (FTIR-6800, JASCO, Max resolution 0.07 cm^− 1^, Japan) in the range from 500 to 4000 cm^− 1^ and transmission electron microscopy (TEM, JEM-2100 F, 200 kV FE, TEM resolution as high as 0.19 nm, JEOL). The UV-Vis absorption spectrum was measured by a UV-Vis spectrophotometer (2600, Shimadzu, Japan) in the wavelength range from 200 to 1400 nm, and photoluminescence was examined by a fluorescence spectrophotometer (Hitachi F-2700 FL, Japan, 150 W Xenon lamp) for different excitation wavelengths ranging from 320 to 400 nm.

The surface of N-CQDs was examined by the X-ray photoelectron spectroscopy (XPS) using a photoelectron spectrometer (Themo Fisher Scientific, USA) with Al K_α_ at 1350 eV. The surface charge of CQDs was estimated by Zeta potential using the phase analysis light scattering (PALS) method (NanoBrook Zeta Potential Analyzer, USA) with a scattering angle of 15º and Zeta potential range (-500 to 500 mV). Cytotoxicity of the N-CQDs synthesized against WI-38 cell lines was studied by the standard MTT assay^21^ (see Sect. 2.2). Antimicrobial activity of N-CQDs against four different types of bacteria and one type of fungi was measured as shown in Sect. 2.3.

### MTT assay

The cytotoxic concentration (IC_50_), which is the concentration required to cause toxic effects in 50% of intact cells, it was investigated for N-CQDs in WI-38 cell lines using MTT assay. WI-38 cell lines (purchased from Tissue Culture Unit, VACSERA) were cultured in Dulbecco’s modified Eagle’s medium (DMEM) supplemented with 10% phosphate-buffered saline (FBS) and 1% antibiotic (50,000 U/L of penicillin and 50 mg/L of streptomycin). Cultures were kept in T-75 tissue culture flasks at 37 ^°^C in 5% CO_2_ and 95% relative humidity in biochemical incubator. Culture media were replaced at least twice a week. WI-38 were seeded in 96-well microplates to a final concentration of (1 × 10^4^) cells/well containing 100 µL of cell line-specific medium and incubated at 37 ^º^C in a 5% CO_2_ atmosphere. The cells were then treated with N-CQDs at concentrations ranging from (3.9–500 µg/mL) of N-CQDs and incubated at 37 ^º^C for 24 h. Then, the cells were subjected to the MTT analysis to determine cell viability^22^. Optical density was measured at 590 nm on a microplate reader (Sunrise, TECAN, Inc, USA) with control wells containing only cell culture medium. The cytotoxic concentration (IC_50_) was estimated from dose-response curve histograms for each concentration using Graphpad Prism software (San Diego, CA. USA). On the other hand, cell morphology was observed using an inverted microscope (CKX41; Olympus, Japan) equipped with a digital microscopy camera to capture the images representing the morphological changes compared to control cells.

### Antimicrobial activity test

#### In vitro antimicrobial activity test

The most widely used technique for antimicrobial susceptibility testing is the agar disk diffusion test, which gives a quantitative result (zones of inhibition in millimeters) and a qualitative interpretive category. An important advantage of this disk diffusion method is that results can be obtained after 16 to 48 h of incubation^23^. Using sterile medical swabs, the suspension microorganisms were evenly smeared onto Mueller-Hinton (MH) agar medium. Microorganisms species have been used such as *S. aureus*,* MRSA E. coli*,* K. pneumoniae*,* and C. albicans* through the Gram-negative and Gram-positive model pathogens. they were diluted to a concentration of 1.5 × 10^8^ CFU/mL. A 50 µL measure of the diluted microorganisms suspension and 50 µL of different concentrations of N-CQDs were transferred to a 96-well cell culture plate for a final volume of 100 µL/well. Samples of N-CQDs were inoculated in each well at 1000 µg/mL concentration. Then, disks containing a certain amount of samples: N-CQD1, N-CQD2, blank solvent and antibiotic as a control (Gentamycin for bacteria and fluconazole for fungi) were placed on the above medium. For comparison, N-CQDs samples were prepared at 180 °C (N-CQD1) and at 220 °C (N-CQD2) for 24 h, respectively. After incubating the disks in a biochemical incubator for 20 h, the diameter of the inhibition zone around each disk was measured and recorded using a vernier caliper^24^.

#### MIC test

The minimum inhibitory concentration (MIC) test was performed on various microorganisms including: *S.aureus*,* MRSA*,* E.coli*,* K. pneumonia and C. albicans*. Then, these microorganisms were cultured in Luria–Bertani (LB) liquid medium at 37 ºC and under shaking at 180 rpm overnight. The microorganisms were diluted to a concentration of about 1 × 10^6^ CFU/mL. The as-prepared N-CQDs samples have been used with a concentrations derived traditionally from serial twofold dilutions (e.g., 1000, 500, 250, 125, 62.5, . µg/mL). Suspension of microorganism in LB medium without N-CQDs was used as a control, and only LB medium was used as the blank. The mixtures were incubated at 37 ºC for 20 h in the incubator. At the end of incubation, the concentration of microorganisms was determined by measuring the optical density at 600 nm (OD600) using BioTek 800 TS microplate reader^25^.

## Results and discussion

### Structural, morphological, and surface analysis

#### XRD and FTIR analysis

**Fig. 2:**
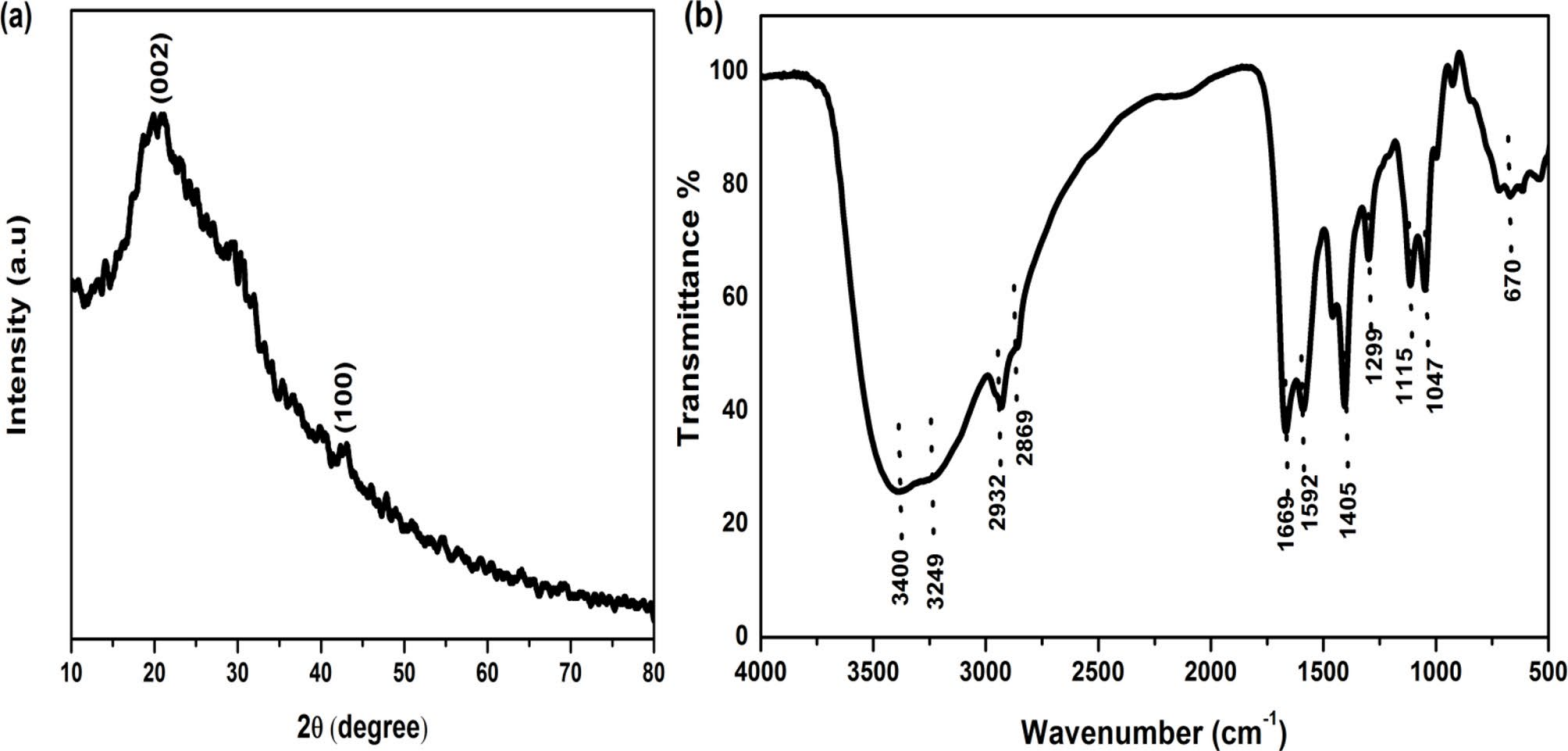
(**a**) XRD pattern and (**b**) FTIR spectrum of the synthesized N-CQDs.

The XRD pattern of the as-prepared CQDs, Fig. 2a shows an intense broad peak centered at 19.9° and the weak peak at approximately 43.1°. These peaks are attributed to the (002) and (100) planes of the graphitic structure of carbon dots, respectively. CQDs have a predominantly graphitic structure with a variable interlayer spacing (d) of 0.4 and 0.25 nm assigned to the (002) and (100) planes, respectively [26]. The variable interlayer spacing which confirms the extreme disorder of carbon atoms may be caused by the introduction of more oxygen-containing groups such as the presence of –COOH and –OH on the surface and edge of CQDs during the hydrothermal treatment to prepare them ^27^. Therefore, XRD results indicate the presence of CQDs with amorphous nature ^28,29^

FTIR spectroscopy was used to identify the functional groups present on the surface of CQDs. The FTIR spectrum of the as-synthesized N-CQDs is shown in Fig. 2b. The observed broad bands at 3400 and 3249 cm^−1^ were attributed to O-H and N–H stretching vibrations, respectively. The bands at 2932 and 2869 cm^−1^ are assigned to the asymmetric and symmetric stretching of the C-H bonds, respectively. The bonds of C = O, C = C, C-N, C-OH and C-O-C functional groups were observed at 1669, 1592, 1299, 1115 and 1047 cm^− 1^, respectively. The absorption band located at 1405 cm^− 1^ is assigned to the bending vibration of O–H bond. Finally, the peak between 900 –670 cm^− 1^ identified the C–H bending in CQDs. The observation of many FTIR bands can be attributed to the heat treatment which tends to facilitate the precursor experiences of dehydration and decomposition, thus forming numerous carbon skeletons^11,30,31^. Therefore, the presence of hydroxyl and carboxylic groups on the surface of N-CQDs becomes logical.

#### TEM analysis

**Fig. 3:**
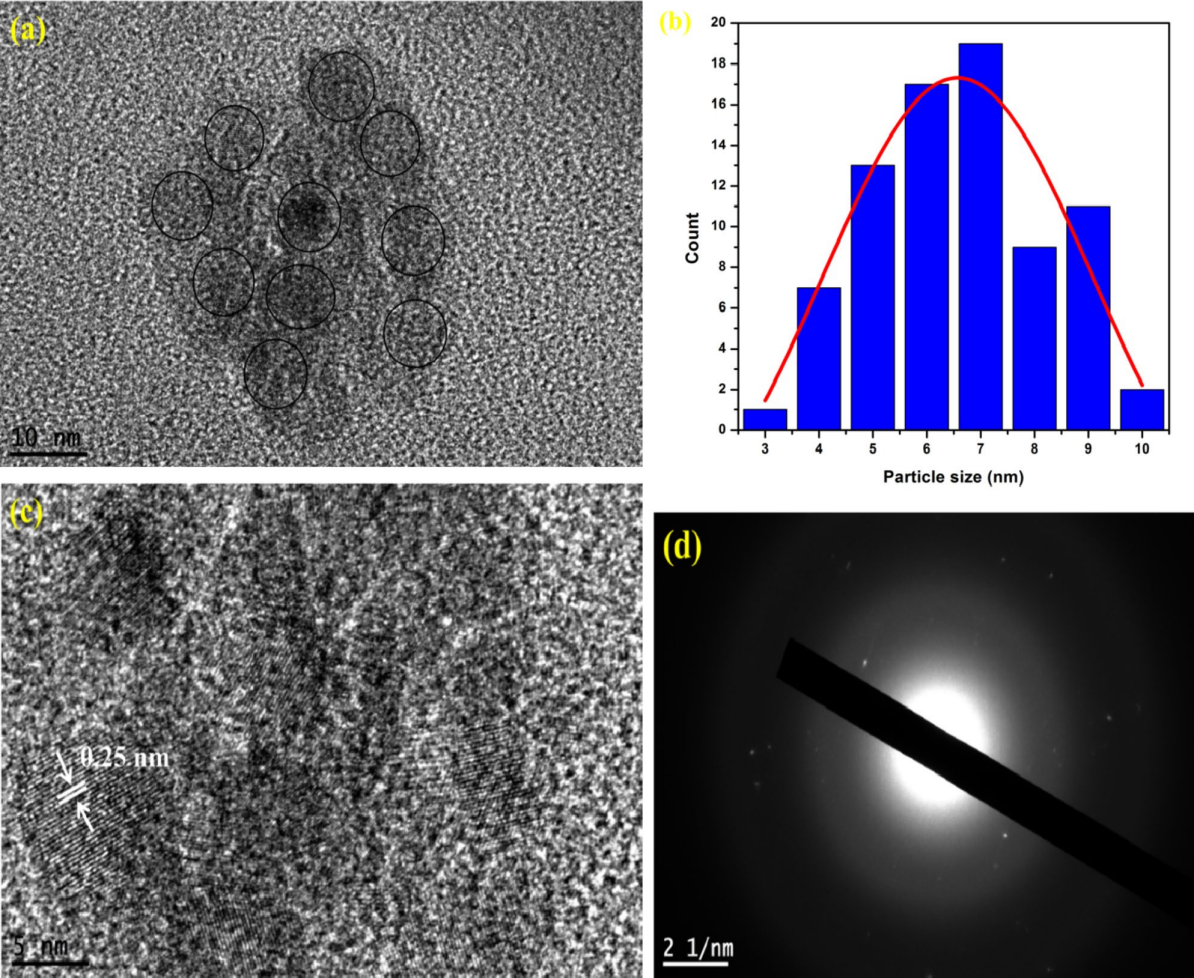
TEM image (**a**) and Gaussian distribution (solid line) of particle size values (**b**) for the as-synthesized N-CQDs. HR-TEM (**c**) and electron diffraction (**d**) images of the synthesized N-CQDs.

Fig. 3 shows the images of the prepared N-CQDs provided by TEM and HR-TEM as well as the particle size distribution. The TEM image reveals that the N-CQDs have a spherical shape with uniform dispersion. Moreover, as shown in Fig. 2b, N-CQDs have an average particle size of 6.57 2.52 nm. The HR-TEM image, Fig. 2c, showed the inter-planer spacing of the CQDs sample is 0.25 nm which is consistent with the graphitic structure of carbon dots ^7,27,28,32^. The electron diffraction pattern, Fig. 3d, confirmed the amorphous nature of the synthesized carbon quantum dots which is consistent with the XRD data ^31^

#### XPS analysis

**Fig. 4:**
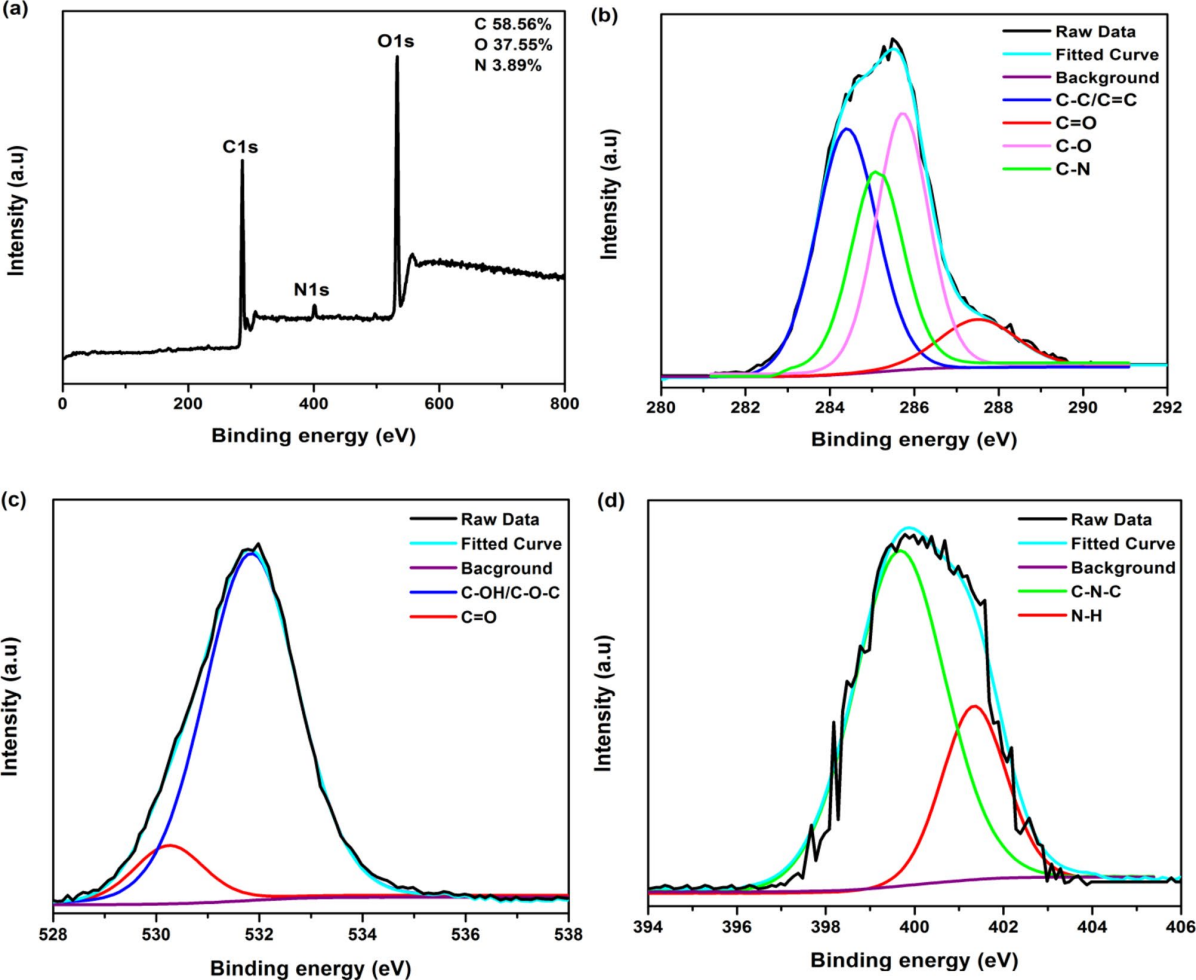
XPS spectra (**a**) survey, (**b**) high-resolution C scan, (**c**) high-resolution O scan and (**d**) high-resolution N scan for of N-CQDs surface.

The XPS survey spectrum as well as the high-resolution C, O and N scan of the surface of N-CQDs is shown in Fig. 4. The XPS survey spectra, Fig. 4a, of the CQDs sample showed three distinct peaks at 286.0, 401.0 and 532.7 eV which are attributed to C1s, N1s, and O1s, respectively. The content of C, N and O elements from the survey spectrum was determined to be 58.6, 3.9 and 37.5%, respectively. In Fig. 4(b), the C1s peak de-convoluted to four peaks at 284.4, 285.0, 285.7, and 287.6 eV originating from C–C/C = C, C–N, C–O and C = O groups, respectively^33,34^. Figure 4c represents O1s spectra with peaks at 530.3 and 531.8 eV which are assigned to the binding energies of C = O and C–O, respectively^35^. The N1s band of CQDs (Fig. 4(d)) can be fitted into two peaks at 399.6 and 401.2 eV, which are assigned to C–N and N–H bonds, respectively. Pyrrolic N formed from the dehydrolysis reaction between carboxyl and amine groups was the major constituent of N in the as-prepared CQDs^36^. The XPS results are consesitent with the results of FTIR measurements and both confirm the presence of oxygen-containing groups and nitrogen-containing groups on the surfaces of the N-CQDs. These hydrophilic functional groups endow the N-CQDs with not only excellent solubility in water but also their fluorescence emission can be highly correlated^37^.

### Optical properties

#### Absorption of N-CQDs solution

**Fig. 5:**
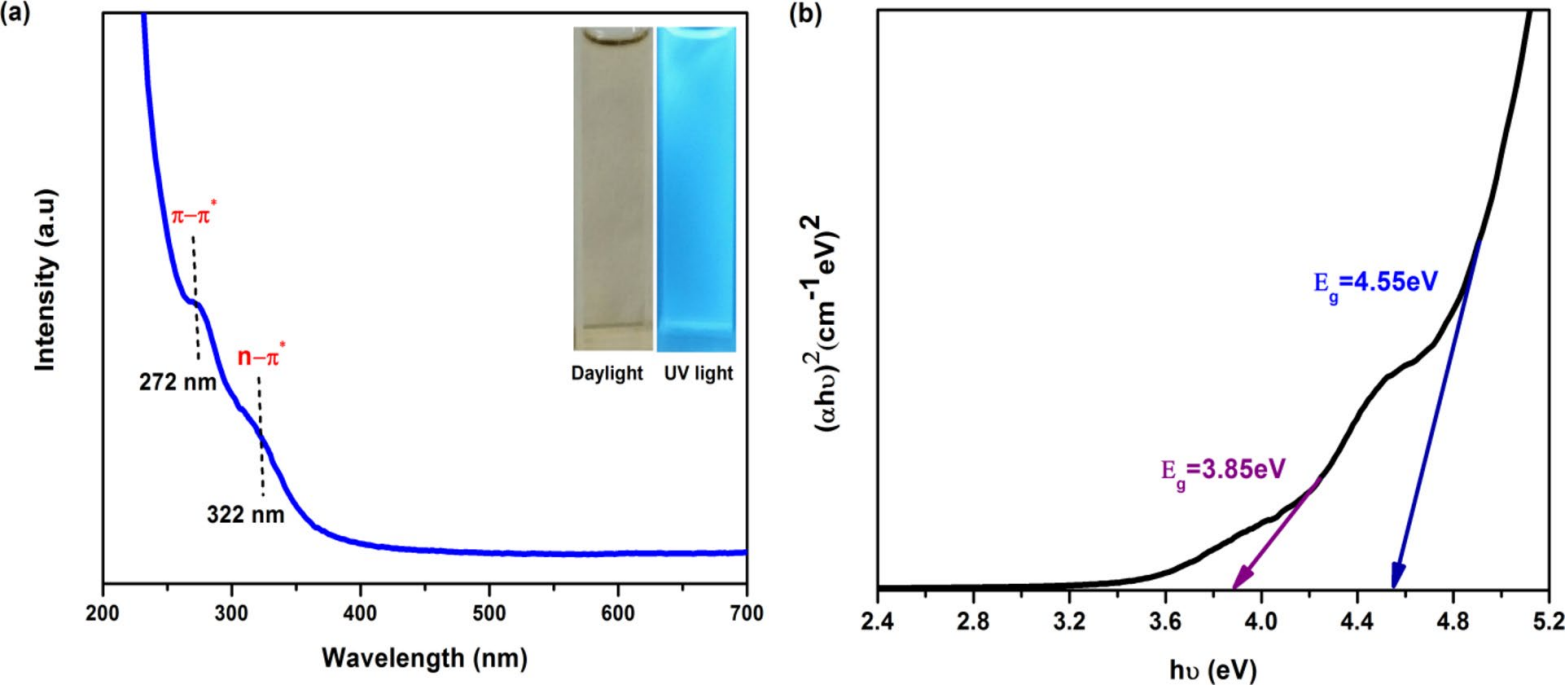
(**a**) UV−Vis absorption spectrum (the insets show the photographs of N-CQDs solution under daylight (left) and 365 nm UV light (right)) and (**b**) Tauc plot curve of N-CQDs.

The UV-Vis absorption spectra of the N-CQDs sample are shown in Fig. 5. Two major absorption bands were observed with absorption maxima at 272 and 322 nm in the UV region. The absorption peaks at 272 and 322 nm are ascribed to the π–π* and n-π* transitions of C = C aromatic bonds and the transition of surface functional groups as C = O, respectively^38,39^. The inset of Fig. 5a shows photographs of N-CQDs under visible daylight (left) and UV (right) illumination. The results reveal that the solution showed bright blue fluorescence under UV irradiation (365 nm) confirming the presence of carbon dots and the pale yellow color of N-CQDs was observed with daylight irradiation. The corresponding optical band gap (*E*_g_) can be estimated from the UV-Vis spectra using a Tauc plot: (αhυ)^2^ versus *hυ*, in which α, *h*, and *υ* are the absorption coefficient, Planck constant, and the light frequency, respectively^40^. Figure 5(b) shows a good linear fitting demonstrating the direct band gap of N-CQDs. The first peak generated around 3.85 eV, corresponds to the unbonded electrons in oxygen and nitrogen dopant atoms in the sp^2^ or sp^3^ carbon^41^. The second absorption at 4.55 eV was the π–π* transition, which is attributed to the sp^2^ electrons in N-CQDs^42^.

#### Fluorescence of N-CQDs solution

**Fig. 6:**
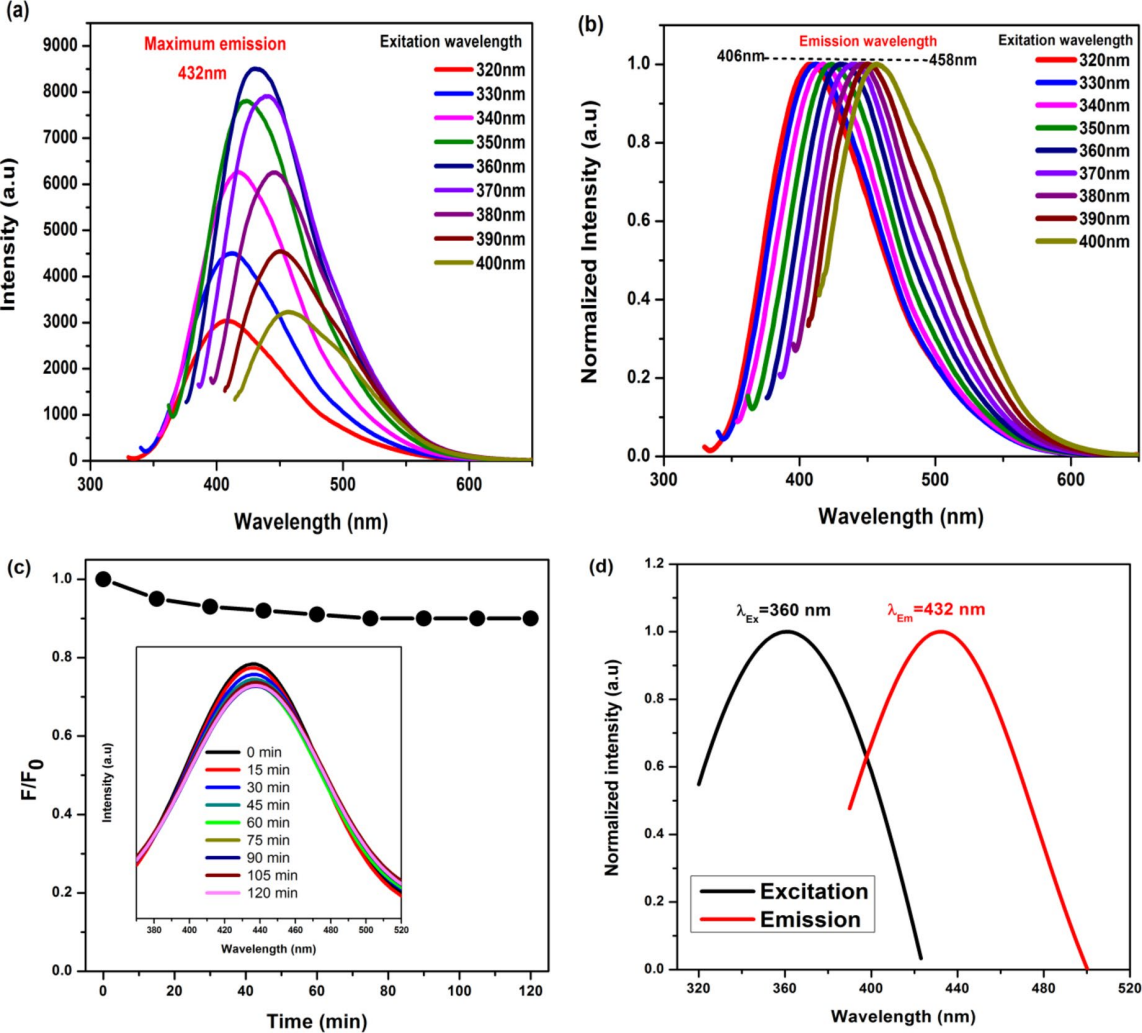
(**a**) PL spectra of as-prepared N-CQDs under different excitation wavelength, (**b**) normalized PL spectra at different excitation wavelengths, (**C**) photostability test of N-CQDs under continuous irradiation of UV-light and (**d**) excitation and emission spectra.

The stability of N-CQDs plays an important role for the different applications. The photostability of N-CQDs was demonstrated through continuous UV light (365 nm) irradiation. The intensity of the emission peak slightly changed at the first than the original intensity (0 min), but after being irritated for 45 min the results suggested that the synthesized N-CQDs showed excellent photostability for a longer time Fig. 6c. This property makes the N-CQDs as good fluorescent probes, which reveals that the N-CQDs from castor seeds have a great potential in practical applications^44,45^.

#### Fluorescence quantum yield (QY) of N-CQDs

The fluorescence quantum yield (QY) of the synthesized N-CQDs was calculated according to this relation^38^:

$$\:Q{Y}_{CQs}=Q{Y}_{R}\left[\frac{\frac{dI}{d{A}_{CDs}}}{\frac{dI}{d{A}_{R}}}\right]\left(\frac{{n}_{CQs}^{2}}{{n}_{R}^{2}}\right)$$

Where QY is the fluorescence quantum yield, *CQDs* and *R* represent the test substance and reference compound. Quinine sulfate dissolved in 0.1 M H_2_SO_4_ (QY_*R*_ = 0.54) was chosen as the reference. n is the refractive index (1.33 for aqueous solution). A is the absorbance at the excitation wavelength of 360 nm. I is the integrated fluorescence intensity under the fluorescence emission spectrum. The quantum yield was found to be 9.6% at excitation wavelength 360 nm^46,47^.

### Zeta potential analysis

**Fig. 7:**
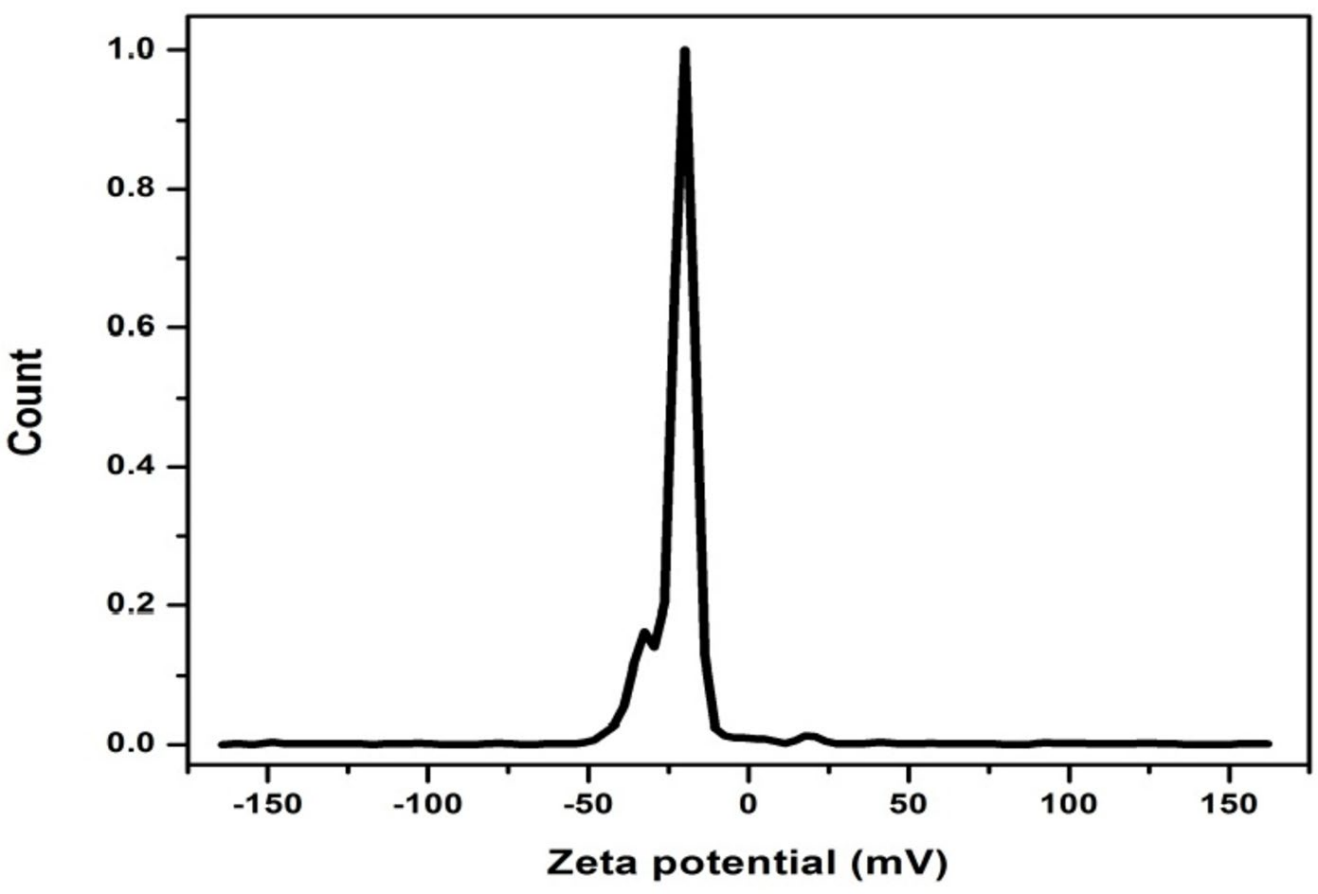
Zeta Potential spectrum of the as-prepared N-CQDs.

The Zeta potential is an important indicator for studying the stability of carbon dots in water by measuring the electrical charge around the CQDs. The large value of zeta potential reflects the high stability of the prepared CQDs^48,49^. Negative values of zeta potential indicate that the surface of the N-CQDs has a negative charge moieties and such moieties are essential to achieving a good dispersion of CQDs in a water-based solvent^48^. The zeta-potential spectrum of the prepared N-CQDs is shown in Fig. 7. The spectrum reveals that the prepared N-CQDs have high stability and good dispersion in their colloidal solutions. This study found a negative zeta-potential value (− 21.86 mV), which was consistent with findings from earlier studies that prepared CQDs from various natural sources^50,51^.

### Biological studies of N-CQDs

#### Cytotoxicity of N-CQDs

**Fig. 8:**
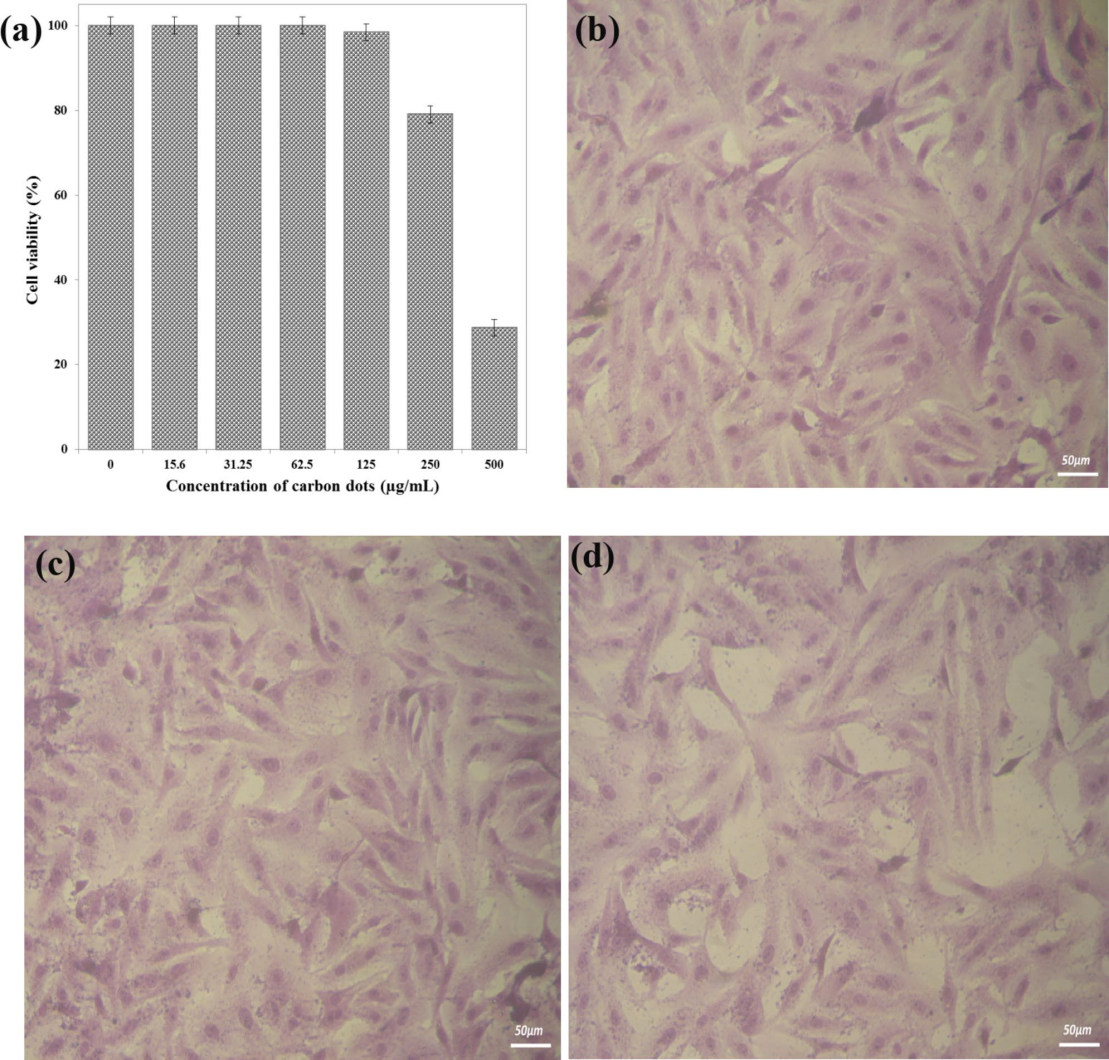
The percentage of cell viability as a function of N-CQDs concentrations (**a**) and morphological images of treated WI-38 cells with the N-CQDs concentration of zero, as a control (**b**), of 100 µg/mL (**c**) and of 500 µg/mL (**d**). Images were taken at (50 μm) scale bar and 200x magnification.

The MTT assay was used to accomplish the viability testing at different N-CQDs concentrations from 3.9 to 500 µg/mL. A graph showing the percentage of cell viability as a function of the N-CQDs concentrations as well as morphological images of WI-38 cells treated with different concentrations of N-CQDs is shown in Fig. 8. Figure 8a shows no change in cell viability until it reaches the cytotoxic inhibitory concentration (IC_50_ = 394.4 ± 13.8 µg/mL). There is no significant loss of normal cell viability of human lung fibroblast (WI-38), even at higher concentrations of N-CQDs. This result confirms that the synthesized N-CQDs show good biocompatibility and are suitable candidates for bioimaging and other biomedical applications. Also, the images captured by the inverted microscope reveal cell inhibition after the treatment with N-CQDs. The cytopathic effects (morphological alterations) were microscopically observed at 200x, as shown in Fig. 8b, c, and d. Other research on the cytotoxicity of CQDs has been conducted, although it has used a variety of cell lines with varying cytotoxic inhibitory concentrations depending on the carbon source^7,11^.

#### Antimicrobial activity and MIC of N-CQDs

**Fig. 9:**
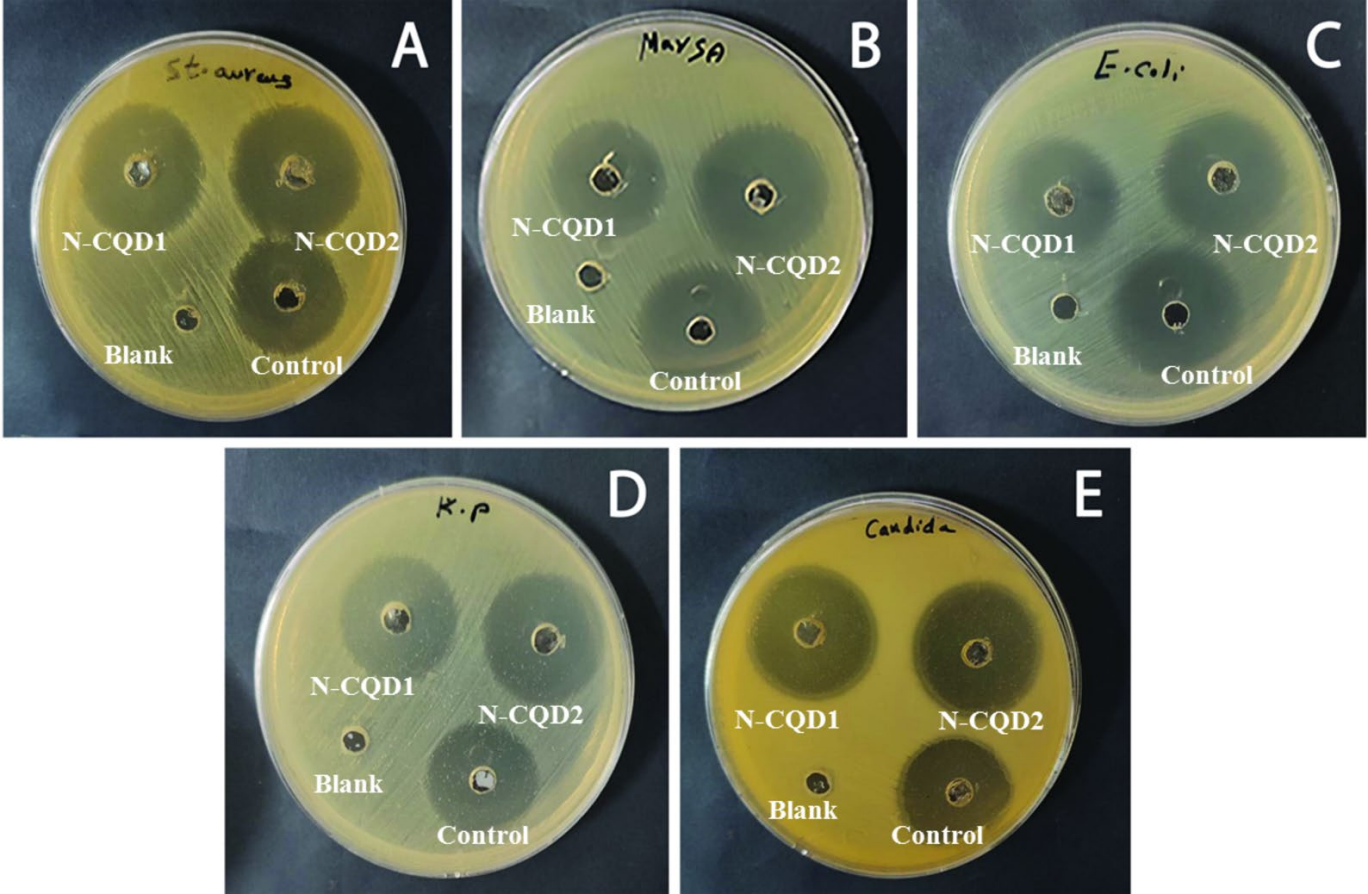
Photographs of culture dishes containing S. aureus (**A**), MRSA (**B**), E. coli (**C**), K. pneumoniae (**D**) and C. albicans (**E**) treated with the prepared N-CQDs. Blank refer to the solvent and control refer to commercial antibiotic.

**Fig. 10:**
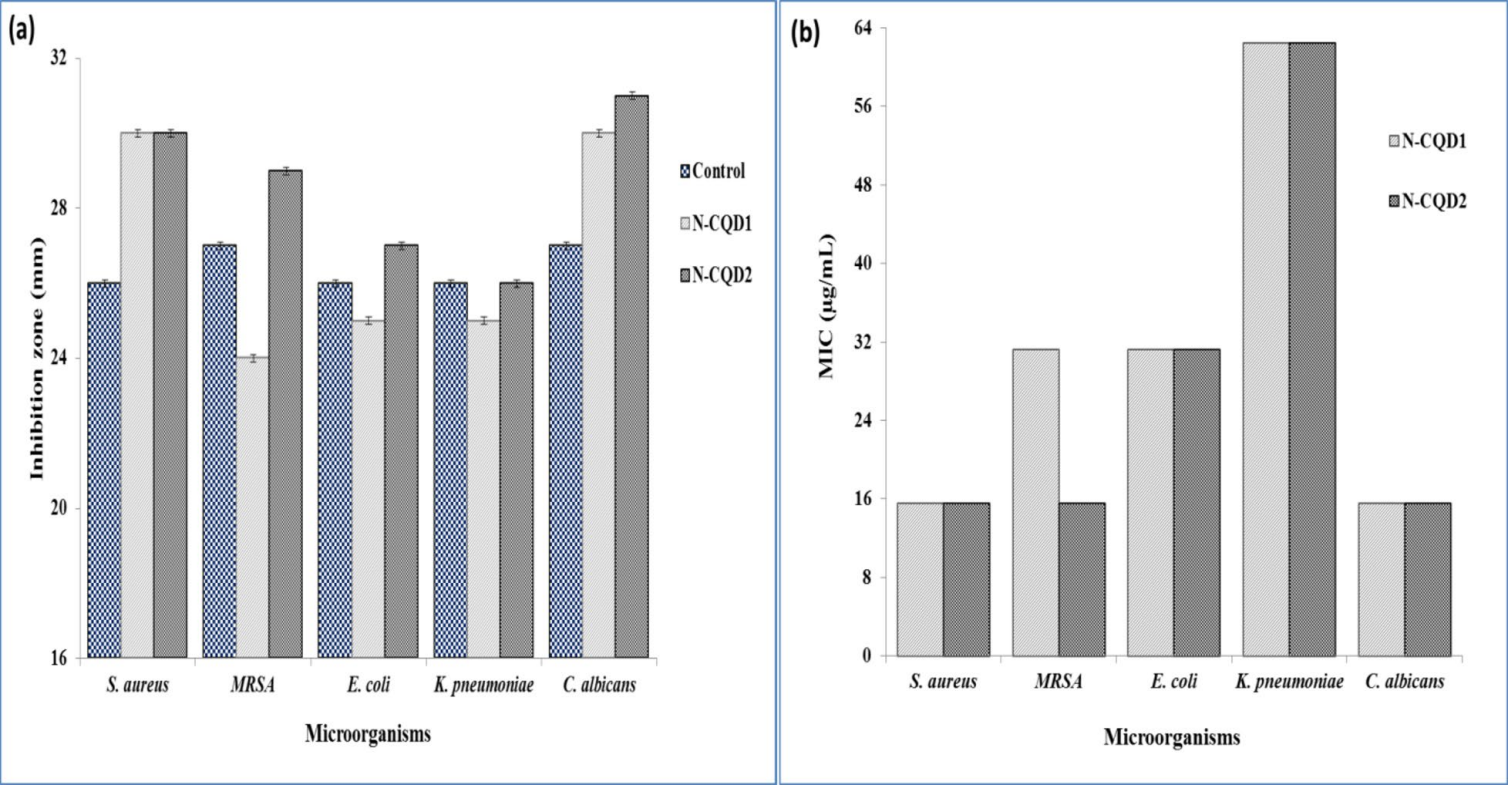
Inhibition effects of N-CQDs on different microorganisms. (**a**) Inhibition zones and (**b**) Minimal inhibition concentration.

The antimicrobial activity of the prepared N-CQDs has been explored with microorganisms such as *S. aureus*,* MRSA E. coli*,* K. pneumoniae*,* and C. albicans* through typical Gram-negative and Gram-positive pathogens. Figure 9 shows images of culture dishes treated with two samples of N-CQDs blank solvent and commercial antibiotic as a control. For comparison, two samples of N-CQDs were prepared at 180 °C (N-CQD1) and at 220 °C (N-CQD2) for 24 h. Table 1; Fig. 10 also represent the values of inhibition zone diameter ​​for four species of bacteria and *C.albicans* fungi after treatment with N-CQD1, N-CQD2 and antibiotic samples. The results showed that the N-CQD1 sample has less effect than the antibiotic on three different bacterial species with a range of 3.9–11.1% decrease in its activity, and its effect is stronger than the antibiotic on *S. aureus* bacteria and *C. albicans* fungi by 15.4 and 11.1%, respectively. On the other hand, the N-CQD2 sample showed enhanced activity compared to the antibiotic for three bacteria species with a range of 3.9–15.8% and for *C.albicans* fungi by 14.8%. For the *K. pneumoniae* species, the diameter value is the same as for culture dishes treated with N-CQD2 and antibiotic. The superior antimicrobial activity of N-CQD2 sample over N-CQD1 sample may be because N-CQD2 sample contains different functional groups and releases of reactive oxygen species (ROS) due to its preparation at different temperatures^52,53^.

**Table 1:** The inhibition zone diameter values ​​for four species of bacteria and *C.albicans* fungi after treatment with N-CQD1, N-CQD2 and antibiotic samples as well as the MIC values for both N-CQDs samples.

Pathogenic microorganisms	Inhibition zone diameter (mm)			MIC (µg/mL)	
	Control (Antibiotic)	*N*-CQD1	*N*-CQD2	*N*-CQD1	*N*-CQD2
*Staphylococcus aureus* (ATCC 6538)	26.0 ± 0.1	30.0 ± 0.1	30.0 ± 0.2	15.62	15.62
Methicillin-resistant* Staphylococcus aureus*	27.0 ± 0.1	24.0 ± 0.1	29.0 ± 0.2	31.25	15.62
*Escherichia coli* (ATCC 8739)	26.0 ± 0.1	25.0 ± 0.2	27.0 ± 0.1	31.25	31.25
*Klebsiella pneumoniae* (ATCC 13883)	26.0 ± 0.1	25.0 ± 0.2	26.0 ± 0.2	62.50	62.50
*Candida albicans * (ATCC 10221)	27.0 ± 0.2	30.0 ± 0.2	31.0 ± 0.1	15.62	15.62

The Minimal inhibitory concentration values ​​for four species of bacteria and *C.albicans* fungi after treatment with N-CQD1 and N-CQD2 samples are shown in Table 1; Fig. 10. The MIC values for three species of bacteria and C.albicans fungi after treatment with N-CQDs1 and N-CQD2 are the same even though they were prepared at different temperatures. Also, the MIC value of the *MRSA* bacteria sample treated with N-CQD1 is doubled compared to that treated with N-CQD2. These results are cosistent with the antibectrial activity results as the diameter of the inhibition zone is the same for all samples expect for this species where its diameter for the sample treated with the N-CQD1 decreased by 17.2% compared to that treated with N-CQD2. The above results showed that N-CQDs prepared at 220 °C had the most effective inhibitory activity against bacteria and fungi. The observed antimicrobial performance can be attributed to different functional groups in N-CQDs that can interfere with cellular enzyme functions and inhibit cellular proliferation. Large π-conjugated N-CQDs arrangements can be easily attached through electron transfer to bacterial and fungi cell walls^54,55^. In addition, the prepared CQDs showed antibacterial activities toward both typical Gram-negative and Gram-positive pathogens due to their release of reactive oxygen species (ROS). Finally, naturally derived CQDs from biomass represent strong candidates as novel antimicrobial agents to combat antibiotic resistance of microorganisms^54,55^.

### CQDs-based fluorescent pH sensor

**Fig. 11:**
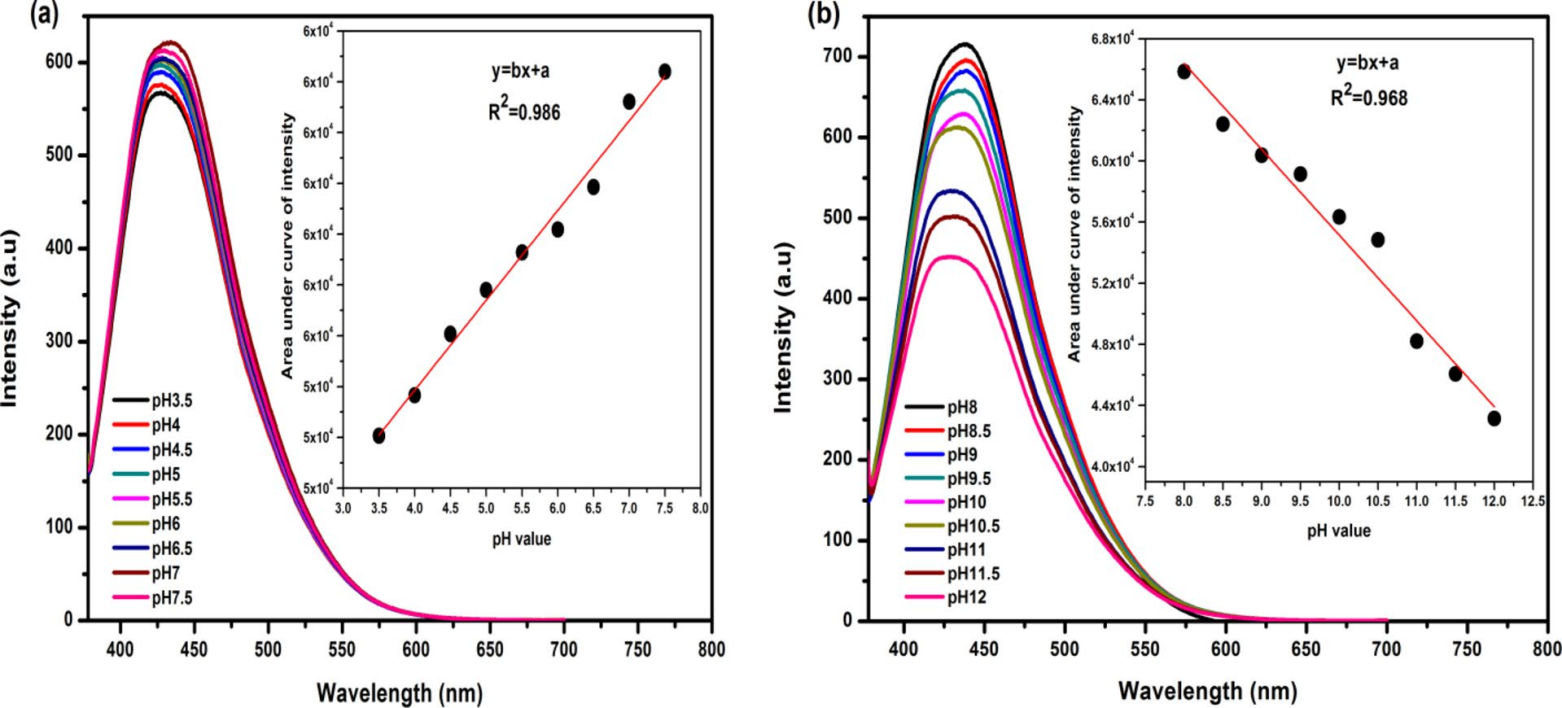
Fluorescence spectra of pristine N-CQDs prepared at a different pH values under the optimal excitation wavelength (360 nm). (**a**) pH value range of 3.5–7.5 and (**b**) pH value range of 8–12. The insets represent linear relationships between the integrated intensity of fluorescence with pH values.

Figure 11 displays the fluorescence spectra of N-CQDs at different pH values. As shown in Fig. 11a, the fluorescence intensity increased significantly in the pH range of 3.5–7.5. Specifically, the inset of Fig. 11a shows the integrated intensity of the fluorescence of N-CQDs versus pH values where there is a linear relationship with the variance R^2^ = 0.986. On the other hand, the fluorescence insanity was observed to decrease strongly with pH value in the range of pH 8–12, as shown in Fig. 11b, where a linear relationship with R^2^ = 0.968 was also found. It should be noted that by adjusting the pH value, the solution of N-CQDs changes from acidic to basic media and the COOH and the OH functional groups on the N-CQDs surface change to COO^-^ and O^-^, respectively, leading to the observation of fluorescence changes (see Fig. 11). Based on these results it can be concluded that the prepared N-CQDs can be used as a high-resolution pH fluorescence probe^6,56,57^.

## Conclusion

In this study, water-soluble nitrogen-doped fluorescent carbon quantum dots were synthesized using castor seeds as the carbon source via a simple and environmentally friendly hydrothermal reaction. The XRD study reveals the amorphous nature of the CQDs in good agreement with TEM analysis, which confirmed the spherical shape of the CQDs with an average particle size of about 6.57 nm. The functional groups on the surface of N-CQDs were confirmed by FTIR spectra as well as surface analysis via XPS spectroscopy. The results showed remarkable optical properties of the prepared N-CQDs as they can emit bright blue fluorescence under UV light. Their excitation-dependent fluorescence behavior and excellent photostability with a quantum yield of about (9.6%) have been successfully used as an effective fluorescent probe. Noticeably, the N-CQDs were also applied to pH sensing in the ranges of 3.5–7.5 and 8–12 under optimum excitation wavelength, where the integrated intensity of the fluorescence of N-CQDs versus pH values demonstrated a linear relationship. The N-CQDs achieve a good dispersion in water due to their negative zeta-potential value of 21.86 mV. Moreover, N-CQDs derived from castor seeds have low cytotoxicity with IC_50_ = 394.4 ± 13.8 µg/mL and effective antimicrobial activity. Therefore, the prepared N-CQDs are suitable candidates for bioimaging and pH sensing applications according to their intense fluorescence, water solubility as well as good dispersibility in aqueous solvents, high stability and good biocompatibility.

## Data Availability

All data generated or analyzed during this study are included in this published article.

## References

[1] Raji, K., Ramanan, V. & Ramamurthy, P. Facile and green synthesis of highly fluorescent nitrogen-doped carbon dots from jackfruit seeds and its applications towards the fluorimetric detection of au 3 + ions in aqueous medium and in in vitro multicolor cell imaging. *New J. Chem.* **43** (29), 11710–11719 (2019).

[2] Wang, Y. & Hu, A. Carbon quantum dots: synthesis, properties and applications. *J. Mater. Chem. C* **2** (34), 6921–6939 (2014).

[3] Tomskaya, A. et al. Optical properties of Tricarboxylic acid-derived carbon dots. *ACS Omega* **7** (48), 44093–44102 (2022). 36506125 10.1021/acsomega.2c05503 PMC9730746

[4] Thirumalai, J. *Quantum dots: recent advances, new perspectives and contemporary applications.* (2023).

[5] Yuan, T. et al. Carbon quantum dots: an emerging material for optoelectronic applications. *J. Mater. Chem. C* **7** (23), 6820–6835 (2019).

[6] Liu, C. et al. A mini review on pH-sensitive photoluminescence in carbon nanodots. *Front. Chem.* **8**, 605028 (2021). 33553104 10.3389/fchem.2020.605028 PMC7862559

[7] Atchudan, R. et al. Sustainable synthesis of carbon quantum dots from banana peel waste using hydrothermal process for in vivo bioimaging. *Phys. E: Low-dimensional Syst. Nanostruct.* **126**, 114417 (2021).

[8] Jung, H. et al. Recent progress on carbon quantum dots based photocatalysis. *Front. Chem.* **10**, 881495 (2022). 35548671 10.3389/fchem.2022.881495 PMC9081694

[9] Kumar, A. et al. Castor seed-derived luminescent carbon nanoparticles for metal ion detection and temperature sensing applications. *Phys. Scr.* **99** (3), 035405 (2024).

[10] Zhang, Y. et al. One-step microwave synthesis of N-doped hydroxyl-functionalized carbon dots with ultra-high fluorescence quantum yields. *Nanoscale* **8** (33), 15281–15287 (2016). 27500530 10.1039/c6nr03125k

[11] Qi, H. et al. Novel N-doped carbon dots derived from citric acid and urea: fluorescent sensing for determination of metronidazole and cytotoxicity studies. *RSC Adv.* **13** (4), 2663–2671 (2023). 36741170 10.1039/d2ra07150a PMC9846458

[12] Atchudan, R. et al. Hydrophilic nitrogen-doped carbon dots from biowaste using dwarf banana peel for environmental and biological applications.*Fuel* **275**, 117821 (2020).

[13] Zheng, L. et al. Electrochemiluminescence of water-soluble carbon nanocrystals released electrochemically from graphite. *J. Am. Chem. Soc.* **131** (13), 4564–4565 (2009). 19296587 10.1021/ja809073f

[14] Xu, X. et al. Electrophoretic analysis and purification of fluorescent single-walled carbon nanotube fragments. *J. Am. Chem. Soc.* **126** (40), 12736–12737 (2004). 15469243 10.1021/ja040082h

[15] Cao, L. et al. Carbon dots for multiphoton bioimaging. *J. Am. Chem. Soc.* **129** (37), 11318–11319 (2007). 17722926 10.1021/ja073527l PMC2691414

[16] Zong, J. et al. Synthesis of photoluminescent carbogenic dots using mesoporous silica spheres as nanoreactors. *Chem. Commun.* **47** (2), 764–766 (2011). 10.1039/c0cc03092a 21069221

[17] Zhu, H. et al. *Microwave synthesis of fluorescent carbon nanoparticles with electrochemiluminescence properties*. *Chem. Commun.* **2009**(34): pp. 5118–5120 . 10.1039/b907612c 20448965

[18] Yang, H. L. et al. Carbon quantum dots: Preparation, optical properties, and biomedical applications. *Mater. Today Adv.* **18**, 100376 (2023).

[19] Magesh, V., Sundramoorthy, A. K. & Ganapathy, D. Recent advances on synthesis and potential applications of carbon quantum dots. *Front. Mater.* **9**, 906838 (2022).

[20] Ovenden, S. P. et al. *Chemical investigations of the castor bean plant Ricinus communis* (Human Protection And Performance Division-Defence Science And …, 2012). DSTO-TR-2786.

[21] Mosmann, T. Rapid colorimetric assay for cellular growth and survival: application to proliferation and cytotoxicity assays. *J. Immunol. Methods* **65** (1–2), 55–63 (1983). 6606682 10.1016/0022-1759(83)90303-4

[22] Mitry, R. et al. *Effects of serum from patients with acute liver failure due to paracetamol overdose on human hepatocytes in vitro*. in Transplantation proceedings. Elsevier. (2005). 10.1016/j.transproceed.2005.03.019 15964424

[23] Espinel-Ingroff, A. et al. Quality control guidelines for amphotericin B, Itraconazole, posaconazole, and voriconazole disk diffusion susceptibility tests with nonsupplemented Mueller-Hinton Agar (CLSI M51-A document) for nondermatophyte filamentous Fungi. *J. Clin. Microbiol.* **49** (7), 2568–2571 (2011). 21543581 10.1128/JCM.00393-11 PMC3147863

[24] Hao, X. et al. Antibacterial activity of positively charged carbon quantum dots without detectable resistance for wound healing with mixed bacteria infection. *Mater. Sci. Engineering: C* **123**, 111971 (2021). 10.1016/j.msec.2021.111971 33812599

[25] Cockerill, F. R. *Methods for Dilution Antimicrobial Susceptibility Tests for bacteria that grow Aerobically: Approved Standard* (No Title), 2012).

[26] Kumar, A. et al. Green synthesis of carbon dots from Ocimum sanctum for effective fluorescent sensing of Pb2 + ions and live cell imaging. *Sens. Actuators B* **242**, 679–686 (2017).

[27] He, M. et al. Material and optical properties of fluorescent carbon quantum dots fabricated from lemon juice via hydrothermal reaction. *Nanoscale Res. Lett.* **13**, 1–7 (2018). 29882047 10.1186/s11671-018-2581-7 PMC5992114

[28] Ding, H. et al. Facile synthesis of red-emitting carbon dots from pulp-free lemon juice for bioimaging. *J. Mater. Chem. B* **5** (26), 5272–5277 (2017). 32264113 10.1039/c7tb01130j

[29] Alarfaj, N. A., El-Tohamy, M. F. & Oraby, H. F. CA 19 – 9 pancreatic tumor marker fluorescence immunosensing detection via immobilized carbon quantum dots conjugated gold nanocomposite. *Int. J. Mol. Sci.* **19** (4), 1162 (2018). 29641488 10.3390/ijms19041162 PMC5979385

[30] Chandra, S. et al. High throughput electron transfer from carbon dots to chloroplast: a rationale of enhanced photosynthesis. *Nanoscale* **6** (7), 3647–3655 (2014). 24562190 10.1039/c3nr06079a

[31] Shaikh, A. F. et al. Bioinspired carbon quantum dots: an antibiofilm agents. *J. Nanosci. Nanotechnol.* **19** (4), 2339–2345 (2019). 30486995 10.1166/jnn.2019.16537

[32] Yue, X. et al. Green synthesis of fluorescent carbon quantum dots for detection of Hg2+. *Chin. J. Anal. Chem.* **42** (9), 1252–1258 (2014).

[33] Wang, X. et al. Green preparation of fluorescent carbon quantum dots from cyanobacteria for biological imaging. *Polymers* **11** (4), 616 (2019). 30960600 10.3390/polym11040616 PMC6523671

[34] Wang, H. et al. Nitrogen-doped carbon dots for green quantum dot solar cells. *Nanoscale Res. Lett.* **11**, 1–6 (2016). 26781285 10.1186/s11671-016-1231-1 PMC4717124

[35] Edison, T. N. J. I. et al. Turn-off fluorescence sensor for the detection of ferric ion in water using green synthesized N-doped carbon dots and its bio-imaging. *J. Photochem. Photobiol., B* **158**, 235–242 (2016). 26994332 10.1016/j.jphotobiol.2016.03.010

[36] Yang, P. et al. Microwave-assisted synthesis of xylan-derived carbon quantum dots for tetracycline sensing. *Opt. Mater.* **85**, 329–336 (2018).

[37] Velo-Gala, I. et al. Activated carbon as photocatalyst of reactions in aqueous phase. *Appl. Catal. B* **142**, 694–704 (2013).

[38] Bakier, Y., Ghali, M. & Zahra, W. Highly sensitive fluorescent detection of pyridine using small size carbon quantum dots derived from folic acid. *J. Phys. D* **53** (40), 405103 (2020).

[39] Aly, A. et al. Non-synthetic luminescent graphene quantum dots in coconut water for aniline sensing applications. *Mater. Res. Bull.* **171**, 112603 (2024).

[40] Hsu, H. C. et al. Graphene oxide as a promising photocatalyst for CO 2 to methanol conversion. *Nanoscale* **5** (1), 262–268 (2013). 23160369 10.1039/c2nr31718d

[41] He, S. et al. Band structures of blue luminescent nitrogen-doped graphene quantum dots by synchrotron-based XPS. *Surf. Sci.* **676**, 51–55 (2018).

[42] Kroupa, D. M. et al. Tuning colloidal quantum dot band edge positions through solution-phase surface chemistry modification. *Nat. Commun.* **8** (1), 15257 (2017). 28508866 10.1038/ncomms15257 PMC5440806

[43] Zhu, S. et al. The photoluminescence mechanism in carbon dots (graphene quantum dots, carbon nanodots, and polymer dots): current state and future perspective. *Nano Res.* **8**, 355–381 (2015).

[44] Atchudan, R. et al. Betel-derived nitrogen-doped multicolor carbon dots for environmental and biological applications. *J. Mol. Liq.* **296**, 111817 (2019).

[45] Guo, Y. et al. Thermal treatment of hair for the synthesis of sustainable carbon quantum dots and the applications for sensing Hg2+. *Sci. Rep.* **6** (1), 35795 (2016). 27762342 10.1038/srep35795 PMC5071893

[46] Atchudan, R. et al. Highly fluorescent nitrogen-doped carbon dots derived from Phyllanthus acidus utilized as a fluorescent probe for label-free selective detection of Fe3 + ions, live cell imaging and fluorescent ink. *Biosens. Bioelectron.* **99**, 303–311 (2018). 28780346 10.1016/j.bios.2017.07.076

[47] Atchudan, R. et al. Tunable fluorescent carbon dots from biowaste as fluorescence ink and imaging human normal and cancer cells. *Environ. Res.* **204**, 112365 (2022). 34767820 10.1016/j.envres.2021.112365

[48] Dager, A. et al. Synthesis and characterization of mono-disperse carbon quantum dots from fennel seeds: photoluminescence analysis using machine learning. *Sci. Rep.* **9** (1), 14004 (2019). 31570739 10.1038/s41598-019-50397-5 PMC6769153

[49] Samadian, S. et al. A novel alginate-gelatin microcapsule to enhance bone differentiation of mesenchymal stem cells. *Int. J. Polym. Mater. Polym. Biomater.* **71** (6), 395–402 (2022).

[50] Ateia, E. E., Rabie, O. & Mohamed, A. T. Assessment of the correlation between optical properties and CQD preparation approaches. *Eur. Phys. J. Plus* **139** (1), 24 (2024).

[51] Chai, S. et al. P-doped carbon quantum dots with antibacterial activity. *Micromachines* **12** (9), 1116 (2021). 34577758 10.3390/mi12091116 PMC8466419

[52] Deme, G. et al. *Effect of hydrothermal reaction temperature on fluorescent properties of carbon quantum dots synthesized from lemon juice for adsorption applications.* Journal of Nanomaterials, 2023: pp. 1–10. (2023).

[53] Hussen, N. H. et al. Carbon dot based carbon nanoparticles as potent antimicrobial, antiviral, and anticancer agents. *ACS Omega* **9** (9), 9849–9864 (2024). 38463310 10.1021/acsomega.3c05537 PMC10918813

[54] Pandiyan, S. et al. Biocompatible carbon quantum dots derived from sugarcane industrial wastes for effective nonlinear optical behavior and antimicrobial activity applications. *ACS Omega* **5** (47), 30363–30372 (2020). 33283084 10.1021/acsomega.0c03290 PMC7711700

[55] Rajendiran, K. et al. Antimicrobial activity and mechanism of functionalized quantum dots. *Polymers* **11** (10), 1670 (2019). 31614993 10.3390/polym11101670 PMC6835343

[56] Wang, L. et al. *Rationally Designed Efficient dual-mode colorimetric/fluorescence Sensor Based on Carbon dots for Detection of pH and Cu2 + ions*6p. 12668–12674 (ACS sustainable chemistry & engineering, 2018). 10.

[57] Zhang, Y., Cheng, S. & Zhang, Y. Green fluorescent carbon dots for sensing of quercetin and pH and cell imaging. *Luminescence* **39** (2), e4638 (2024). 10.1002/bio.4638 38083837

